# Classification of Plant Ecological Units in Heterogeneous Semi-Steppe Rangelands: Performance Assessment of Four Classification Algorithms

**DOI:** 10.3390/rs13173433

**Published:** 2021-08-29

**Authors:** Masoumeh Aghababaei, Ataollah Ebrahimi, Ali Asghar Naghipour, Esmaeil Asadi, Jochem Verrelst

**Affiliations:** 1 Department of Range and Watershed Management, Faculty of Natural Resources and Earth Sciences, Shahrekord University, 8818634141 Shahrekord, Iran; 2 Image Processing Laboratory (IPL), Parc Científic, Universitat de València, 46980 Paterna, València, Spain

**Keywords:** object-based classification, machine learning algorithms, principal component analysis, plant ecological units mapping

## Abstract

Plant Ecological Unit’s (PEUs) are the abstraction of vegetation communities that occur on a site which similarly respond to management actions and natural disturbances. Identification and monitoring of PEUs in a heterogeneous landscape is the most difficult task in medium resolution satellite images datasets. The main objective of this study is to compare pixel-based classification versus object-based classification for accurately classifying PEUs with four selected different algorithms across heterogeneous rangelands in Central Zagros, Iran. We used images of Landsat-8 OLI that were pan-sharpened to 15 m to classify four PEU classes based on a random dataset collected in the field (40%). In the first stage, we applied the following classification algorithms to distinguish PEUs: Minimum Distance (MD), Maximum Likelihood Classification (MLC), Neural Network-Multi Layer Perceptron (NN-MLP) and Classification Tree Analysis (CTA) for pixel based method and object based method. Then, by using the most accurate classification approach, in the second stage auxiliary data (Principal Component Analysis (PCA)) was incorporated to improve the accuracy of the PEUs classification process. At the end, test data (60%) were used for accuracy assessment of the resulting maps. Object-based maps clearly outperformed pixel-based maps, especially with CTA, NN-MLP and MD algorithms with overall accuracies of 86%, 72% and 59%, respectively. The MLC algorithm did not reveal any significant difference between the object-based and pixel-based analyses. Finally, complementing PCA auxiliary bands to the CTA algorithms offered the most successful PEUs classification strategy, with the highest overall accuracy (89%). The results clearly underpin the importance of object-based classification with the CTA classifier together with PCA auxiliary data to optimize identification of PEU classes.

## 1 Introduction

Accurate vegetation maps of the distribution and extent of vegetation communities across heterogeneous landscapes are invaluable for management of landscape changes and conservation planning [1], natural resources [2] and ecosystem services [3]. Vegetation community maps also provide important information related to changes in environmental conditions, climate and biodiversity studies [4,5]. Land cover assessment and classification for quantitative detection of sparse vegetation and production of vegetation maps are important tasks in optical remote sensing [6]. Due to field data limitations and temporal inconsistencies of field datasets, the needed vegetation maps for management and monitoring are often updated only every few years [7]. Plant Ecological Units (PEUs), being the fundamental unit of natural habitats, are defined as frequently co-occurring plant species that differ from other areas in the ability to produce mixtures of plant species [8]. Moreover, PEUs are considered as the ecological units, which similarly respond to management actions and natural disturbances [9]. Therefore, PEUs are commonly considered relevant indicators of ecosystem services, mapping ecosystem services [10] and assessing the conservation status of a site [11].

Although the concept of PEUs in rangeland monitoring and assessment is generally accepted, the importance and benefits of PEUs monitoring are poorly understood. Over four decades of satellite remote sensing application to assessment and vegetation mapping using satellite image processing for quantitative detection of sparse vegetation, distinction of PEUs remains problematic and challenging [12]. Generally, PEUs behave spectrally alike and due to low inter-class separability, form complex spatial structures within the heterogeneous landscape [13]. The production of reliable and accurate PEUs maps in heterogeneous landscapes is typically based on the classification of raw satellite imagery. Yet, these heterogeneous vegetation communities impose challenges to spectral classifier methods [14,15]. In order to optimize classification of PEUs from optical data, the selection of an ideal classification method needs to be considered. Previous studies indicated that the selection of the best classifier methods depends mainly on the satellite images, characteristics of the study area, the classification system and selected algorithm performance [16]. When medium spatial resolution imagery (e.g., Landsat images) is used for the classification of large areas, the approach of analyzing pixels individually can produce misclassifications. Especially in PEUs with spectral similarity, because in pixel-based methods each pixel is classified into only one class based on the digital values. Consequently, pixel-based approaches may produce poor classification results. One possible solution is using the Object Based Classification Method (OBCM) [17]. In this way, image segmentation merges pixels into objects, and classification is conducted based on the objects instead of an individual pixel [18]. Many classifiers from statistical-based approaches such as MD (Minimum Distance) and MLC (Maximum Likelihood Classification) to artificial intelligence and machine learning approaches such as ANN (Artificial Neural Network), RF (Random Forest), and DT (Decision Tree) are commonly used for vegetation classification [16,19,20], whereby machine learning methods increasingly replace traditional statistical analysis methods. For instance, a variety of machine learning algorithms were applied to land cover mapping across a range of spatial scales [21,22]. Such algorithms can substantially reduce the cost and time of constructing land cover maps. At the same time, several studies recommended incorporating auxiliary spatial data to help distinguish land cover features from satellite images [5,23].

Altogether, this study aims to compare pixel-based and object-based approaches with selected four different classifier algorithms for PEUs mapping. Two specific objectives were addressed: (1) the first stage aimed to evaluate the use of Minimum Distance (MD), Maximum Likelihood Classification (MLC), Neural Network-Multi Layer Perceptron (NN-MLP) and Classification Tree Analysis (CTA) algorithms for pixel- and objectbased methods Fj to identify PEUs. (2) In the second stage, auxiliary data (PCA) were used to make the most accurate PEUs classification map based on the best performing algorithm. This study will eventually provide insights into the classification strategies for the PEUs mapping in the semi-arid rangelands.

## 2 Materials and Methods

### 2.1 Study Area

**Figure 1:**
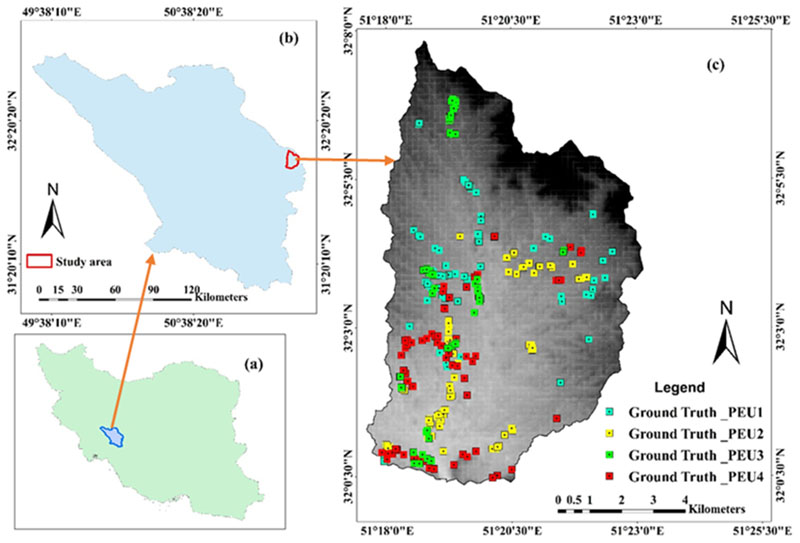
The location of the study area (**a**)—Iran border; (**b**) — Central Zagros border; and (**c**)—Study area border (Marjan). A set of sampling points of PEUs recorded in the field which later divided into two groups of training (40%) for classification and ground truth (60%) for accuracy testing.

Semi-steppe rangelands Marjan is located within the Central Zagros in Southwest, Iran. The area covers 7736.24 ha extending from 51°18′53″ to 51°19′12″E and 32°03′56″ to 32°04′05″N and with a mean elevation of 2697.48 m a.s.l (Figure 1). The climate is arid (temperate and cold winters, and warm and dry summers), characterized by an average annual rainfall (1988–2018) of 220 mm. Various PEUs thrive in the study area. Despite its low average annual rainfall (220 mm), due to appropriate implementing management practices, much of the area has suitable vegetation conditions, whereby perennial grasses and shrubs dominate. PEUs can be straightforwardly observed in this study area due to narrow ecotones and relatively sharp borders between them.

### 2.2 Field Measurements of PEU

Four PEU classes were identified in the study area (Table 1), namely: (1) PEU1 (*Astragalus verus* Olivier (As ve)), (2) PEU2 (*Bromus tomentellus* Boiss (Br to)), (3) PEU3 (*Scariola orientalis* Sojak (Sc or)), and (4) PEU4 (*Astragalus verus* Olivier—*Bromus tomentellus* Boiss (As ve—Br to)). Canopy cover data could potentially be used to identify PEUs from structural, compositional or combination of both, the so-called physionomic-floristic classification, to have a sound and accurate perspective on PEUs. We sampled the four identified PEUs using three replicates, in each of which canopy cover was sampled along three transects of 100 m that were evenly distributed throughout the Marjan. The sampling was systematic-randomly (the first node was selected systematically but the rest were randomly distributed along the transects). We collected species-based canopy cover within each quadrat. In each PEU, canopy cover percentage was calculated and the PEU types were named according to their dominant floristic composition. For this purpose, first, the dominant plant species of each PEU was identified and then its accompanying species was determined with having 50% or more canopy cover of previously dominant species cover. Thus, each PEU was named based on a physiognomic–floristic method.

### 2.3 Remotely Sensed Data

We downloaded ortho-rectified (L1T) Landsat Operational Land Imager (OLI) images acquired on 10 June 2018. The image location corresponds with path 164, row 38 from the USGS (https://earthexplorer.usgs.gov/). This date almost represents a peak in the phenological development for the majority of PEUs in the area. Bands uninformative for vegetation mapping (cirrus, coastal aerosol and thermal-TIR bands) were excluded [24]. The Dark Object Subtraction method (DOS) was used to obtain image surface reflectance and to remove the atmospheric effects [25]. This method is a simple atmospheric correction that subtracts the lowest image digital values (DN) from all other DN values across an image. In other words, DOS subtracts the minimum DN existing in the image, considering it as the constant path irradiance (mostly in the pure water bodies), which is assumed to be systematically distributed over the image.

Since, in this study, we aimed at merely distinguishing the qualitative value of PEUs (not, for instance, their quantitative forage yield that is more dependent on the image correction method), therefore, DOS was found appropriate to apply over the image [2]. Finally, the study area boundary was cut using the digital border map of the area.

### 2.4 Methodology

#### 2.4.1 Multispectral Image’s Pan Sharpening

The Landsat OLI-8 images spatial resolution vary from 30 m (multispectral bands) to 15 m (panchromatic band). By having the opportunity to exploit the panchromatic band (having a wider spectral wavelength and higher spatial resolution) and consequently enhancing the possibility to discern PEUs border, multispectral bands of the 30 m resolution were pan-sharpened using the corresponding panchromatic band of 15 m spatial resolution. Atmospherically corrected raw bands of the multispectral Landsat-8 data (bands 2–7) were stacked into a set of datasets and named “Raw bands”.

#### 2.4.2 Sampling PEUs and Classification System

After distinguishing dominant PEUs within the study area, for each identified PEUs 75 sample sites were observed and recorded by field excursion. The XY position of each representative PEUs points were recorded using a Garmin eTrex 32× Handheld GPS. In total, 300 samples were recorded for the four PEUs (Figure 1). The sample sites were then divided into two groups of 120 sites (40%) used for classification as the “training sites” and 180 sample sites (60%) used for the validation of classification results as the “testing sites”.

#### 2.4.3 PEUs Classification Using Different Classification Algorithms

The goal of this study was to determine the most accurate PEU classification strategy by evaluating the accuracy of pixel- and object-based classifications using distinct classification algorithms. Although multiple classification approaches can be applied, the one that provides superior classification results for a specific study area and different factors mapping (land cover) is yet to be deduced. Four distinct classifiers were selected due to their different conceptual and mathematical designs, being: (1) Minimum Distance (MD), (2) Maximum Likelihood Classification (MLC), (3) Neural Network-Multi Layer Perceptron (NN-MLP), and (4) Classification Tree Analysis (CTA). These algorithms are briefly outlined below.

MD undertakes a Minimum Distance to mean classification of data based on the information contained in a set of signature files. The MD to means classification is based on the mean reflectance on each band for a signature. Pixels are assigned to the class with the mean closest to the value of that pixel [26].

MLC is a traditional and statistical-based algorithm that due to its robustness and its availability is widely used in remote sensing classification applications [15]. In this classification algorithm pixels are determined to the most likely class based on a comparison of the posterior probability that it belongs to each of the signatures being considered [27].

Neural networks are an interconnected group of nodes. Each node represents an artificial neuron with a connection from the output of one neuron to the input of another. Using the training dataset, weights are established for each neuron and the model is able to capture the non-linear relationships of the model. One of the most popular neural networks used in remote sensing is the feed-forward Multi-Layer Perceptron (MLP) neural network trained via backpropagation (BP) algorithm. The results are based on information from training sites. MLP also performs a regression analysis between input variables and one dependent variable with the output containing one output neuron, i.e., the predicted memberships. A typical MLP network contains one input layer, one output layer, and one or more hidden layers (though, one hidden layer is generally adequate for most problems) [6,28].

Classification Tree Analysis (CTA) successively splits training data to form homogenous subsets resulting in a hierarchical tree of decision rules. In other words, the CTA algorithm is a top-down inductor of decision trees that expands nodes in depth-first order for each step using the divide-and-conquer strategy [29].

#### 2.4.4 Segmentation

In the object-based approach, the main step is to prepare image data apt for segmentation. In our research, pan-sharpened multispectral bands of Landsat-8 images (as raw reflectance bands) were partitioned into homogeneous objects using a segmentation algorithm in TerrSet ver.18, software. Segmentation groups adjacent pixels into image segments according to their spectral similarity and creates highly homogeneous image objects while minimizing the average heterogeneity at an arbitrary resolution [30]. Segmentation employs a divide delineation strategy to partition input imagery based on their variance. A derived variance image is treated as a surface image allocating pixels to particular segments based on variance similarity. The objects created can be used as the base of the map classification and other processing procedures. During the segmentation process, the weighting of input data and parameters such as weight mean and the variance factors must be controlled for evaluating the similarity between neighboring segments. In addition, the width and height of the moving window from which the module will derive a variance image of each layer and similarity tolerance must be specified [31]. To obtain the segmentation parameters with the greatest precision for each study, their ability to delineate PEU in various scenarios must be evaluated. Several parameters were initially tested: the most satisfactory combination for weight mean factor, weight variance factor, window width and similarity tolerance values were set as 0.5, 0.5, 3 and 5, respectively.

#### 2.4.5 Auxiliary Data and Prediction Assessment

After selecting the most accurate algorithms identified in the previous stages, PCA bands (first three principal components) were subsequently incorporated to arise the accuracy of PEUs classification process. This approach is intended to optimize the classification accuracy in the sparsely vegetated arid rangeland with relatively similar reflectance. These PCAs were generated using the spectral information and explained >99% of the data variation.

For each classification process, the mapping accuracy was evaluated by means of the confusion matrix resulting from crossing ground truth image of “testing sites” and outcome map of classification process. We built confusion matrices and estimated Kappa Index of Agreement (KIA), Overall Accuracy (OA), Producer’s Accuracy (PA), and User’s Accuracy (UA). The producers and users’ accuracies for individual PEU classes were compared in order to understand the best accuracy of the PEUs classes using auxiliary data (PCA). The OA is determined by dividing the total number of correctly classified pixels and indicates the percentage of correctly classified pixels. The Kappa coefficient usually serves to assess the statistical difference between classifications that indicate a more conservative estimation than simple percent agreement value. The Equations used to calculate the KIA (1), OA (2), PA (3) and UA (4) are given as follows:

(1) $$\;(\text{KIA})=\frac{\sum_{i=1}^{k}X_{ii}-\sum_{i=1}^{r}X_{i}+X_{+i}\;\;\;}{n^{2}-\sum_{i=1}^{r}X_{i}+X_{+i}}$$

(2) $$\text{OA}=\frac{\sum_{i=1}^{k}X_{ii}}{N}$$

(3) $$\text{PAj}=\frac{X_{j,j}}{\sum_{j=1}^{n}X_{i,j}}$$

(4) $$\text{UAi}=\frac{X_{i,i}}{\sum_{j=1}^{n}X_{i,j}}$$

where *n* is the total number of all classifications; X*ij*, is an element, located at *i*th row and *j*th column of the confusion matrix (or error matrix); PA*j* represents PA of class *j*, and UA*i* represents UA of class *i*.

#### 2.4.6 Statistical Comparison of Classification Algorithms

As the confusion matrix only gives the performances of PEUs maps based on validation samples, we additionally computed the Friedman test. This test enabled us to assess whether there was a statistically significant difference between different classification algorithms when using either pixel- or object-based approaches.

**Figure 2:**
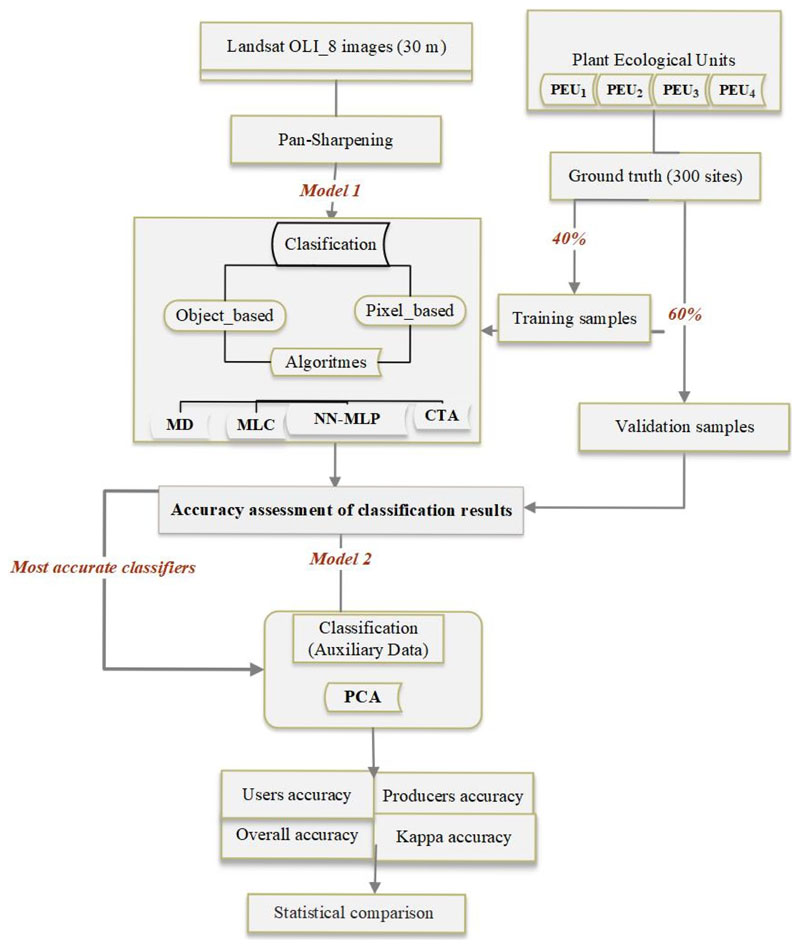
Workflow of mapping PEUs through combining images classification methods with classification algorithms.

Figure 2 shows the conducted workflow to assess the effects of different classification algorithms for PEUs classification accuracy. As depicted in this Figure, firstly multispectral and panchromatic bands of Landsat OLI-8 images were downloaded and pan-sharpened to modify the spatial resolution from 30 to 15 m. Then, by selecting the most accurate algorithms identified in the previous stage, auxiliary data (PCA) were subsequently incorporated in the second step to improve accuracy of PEUs classification process. So, PCA analysis was run to extract the first three principal components (PCAs) of the raw Landsat bands. The collected field samples were split into training (40%) and test data (60%); the first was used for signature development for classification and the latter for accuracy assessment. Finally, the obtained maps from the different classification algorithms were validated using in-field collected test data.

## 3 Results

### 3.1 Pixel-Based Classification

**Figure 3:**
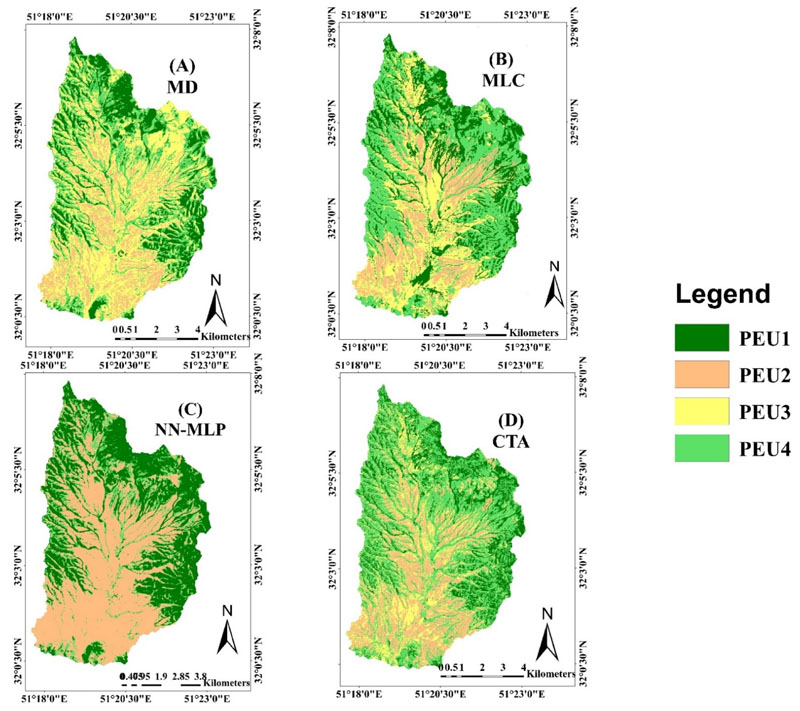
Comparison of pixel-based classification method (**A**) Minimum Distance (MD); (**B**) Maximum Likelihood Classification (MLC); (**C**) Neural Network-Multi Layer Perceptron (NN-MLP); and (**D**) Classification Tree Analysis (CTA). The diagram shows the overall accuracy resulting from classifier performance, which is used in pixel based PEUs classifications.

As shown in Figure 3, the four classifier algorithms led to varying classification results. On the whole, the maps as obtained by MLC, MD, and CTA classifiers showed similar spatial patterns of PEUs. These algorithms showed that PEU1 and PEU4 mostly distributed in margins and sloping of the study area. PEU2 is mostly distributed at the center and flat area, but PEU3 is distributed in almost all regions with varying amounts. The map of the NN-MLP classifier allocated flat areas to PEU2 and steep areas to PEU1 and very little to the PEU4. Nevertheless, PEU3 was removed from the study area. The overall accuracy results of each classifier, which is used in pixel-based PEUs classification. The overall accuracy for MLC, MD, NN-MLP and CTA was 78%, 57%, 55% and 67% respectively. The best pixel-based classification results are from the MLC, and machine learning algorithms NN-MLP and CTA cannot improve overall pixel-based classification accuracies.

### 3.2 Object-Based Classification

**Figure 4:**
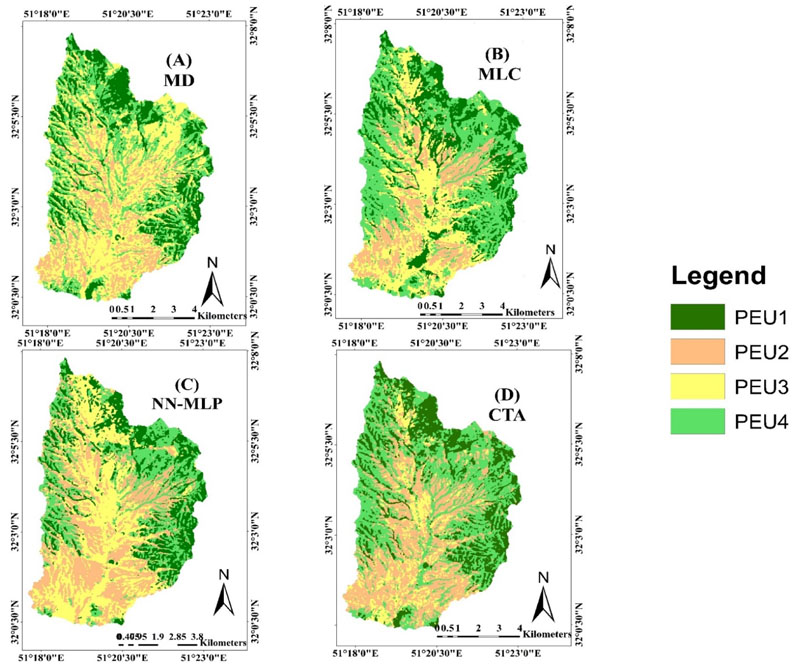
Comparison of Object-based classification method (**A**) Minimum Distance (MD); (**B**) Maximum Likelihood Classification (MLC); (**C**) Neural Network-Multi Layer Perceptron (NN-MLP); and (**D**) Classification Tree Analysis (CTA). The diagram shows the overall accuracy resulting from classifier performance, which is used in Object based PEUs classifications.

Unlike the pixel-based approach, no significant visual difference can be observed between object-based maps in spatial patterns of the PEUs. MLC, MD, NN-MLP and CTA classifiers led to similar spatial patterns of PEUs (Figure 4). All of the algorithms revealed that PEU1 and PEU4 mostly distributed in margins and sloping of the study area, PEU2 mostly distributed at the center and flat areas, and PEU3 almost distributed in all regions. According to the overall PEUs accuracy assessment results among four classification algorithms using the object-based approach, the best overall accuracy of 86% was obtained using the CTA classifier. The overall accuracy for MLC, MD and NN-MLP were 78%, 59% and 72%, respectively.

### 3.3 Comparing Supervised Classification Methods

Accuracy assessments of the PEUs classification using different classification algorithms are shown in Table 2. Of the 8 classification strategies evaluated, except for the MLC algorithm, there is not any significant difference between the object-based and pixelbased approaches. So that in both methods of classification OA is 78%. While in other algorithms (MD, NN-MLP and CTA) the object-based approach outperformed the pixelbased approach.

MLC classification algorithm led to the highest overall kappa and overall accuracy (70% and 78%, respectively) when using the pixel-based approach. Also, in the objectbased approach, the CTA classification algorithm led to highest overall kappa and overall accuracy (80% and 86%, respectively). The results of this algorithm revealed an improvement of the classification accuracy around 8%, 27% and 14% when comparing the MLC, MD and NN-MLP object-based classifiers, respectively.

The overall accuracy for object-based classification with MD, NN-MLP, and CTA algorithms than the pixel-based classification increased with 2%, 17% and 19%, respectively.

### 3.4 Impact of Auxiliary Data on PEUs Classification Accuracy

**Figure 5:**
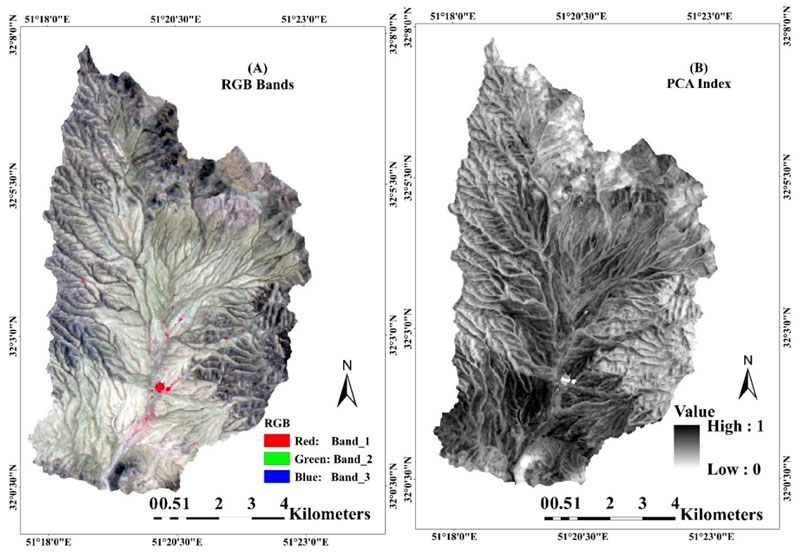
(**A**) Landsat OLI-8 false color-composite (RGB); (**B**) PCA Index, used as auxiliary data to improve accuracy in operational PEUs classification.

While the first step determined the best classification methods and classification algorithms for obtaining the most accurate PEU classification maps, in the second step, PCAs were additionally used as auxiliary data to improve accuracy in operational PEUs classification. The maps are shown in Figure 5. These data include Landsat OLI-8 false color-composite (RGB= bands 3 (green), 4 (red), 5 (infrared)) and the first three principal components of raw bands.

The summary results of confusion matrices for the PEUs classifications achieved from object-based classification algorithms of raw bands (first step) as well as of raw bans and auxiliary data (second step) are presented in Table 3. In this table, producer accuracies, user accuracies, and kappa index of the agreement for each PEUs, overall kappa and overall accuracy of each classification process are reported. When raw bands were applied, PEU4 had the highest UA and KIA with 96 and 95%, respectively. However, PEU2 led to the lowest UA and KIA with 80 and 72%, respectively. The overall kappa was 80 and overall accuracy 86%. Merging the auxiliary data (PCAs) to the raw bands led to the improvement of object-based classification accuracies. The performance of auxiliary data showed that PCA returned the overall kappa accuracy to 85% and overall accuracy 89%. PEU1 led to the highest UA, PA, and KIA with 100, 97 and 100%, respectively, and PEU2 led to lowest UA and KIA with 81 and 74%, respectively when PCAs bands in addition to raw bands were used for classification. The side-by-side comparison of the performance of raw bands and auxiliary data revealed that auxiliary data improved overall accuracy of 5% and overall kappa accuracy of 4% (Table 3).

**Figure 6:**
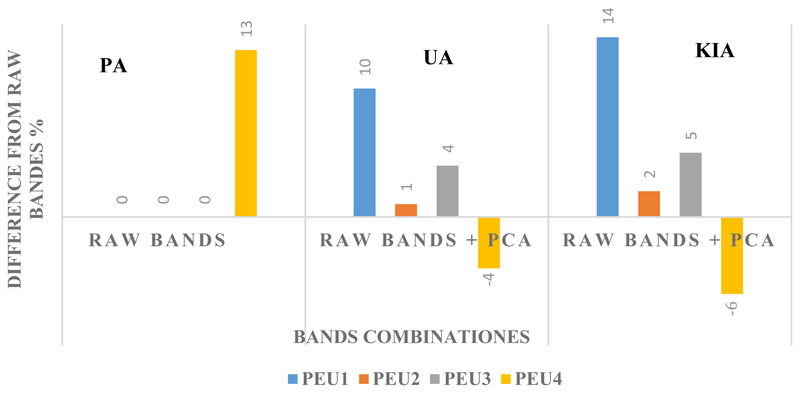
Effects of auxiliary data (PCA) on the accuracy of classification of PEUs. PA: Producer’s Accuracy, UA: User’s Accuracy and KIA: Kappa Index of Agreement.

Figure 6 reveals the effect of auxiliary data (PCAs) from raw bands on the accuracy of classification of PEUs. As shown in this Figure, the classification of most of the PEUs were neutral to auxiliary data while PEU4 significantly profits from the auxiliary data in terms of PA. As indicated in figure, all the PEUs significantly profit from the auxiliary data up to 10%, in terms of UA. Classification of most of the PEUs benefits from auxiliary data up to 14% in terms of KIA; however, the auxiliary data negatively affect KIA of PEU4 by 6%.

**Figure 7:**
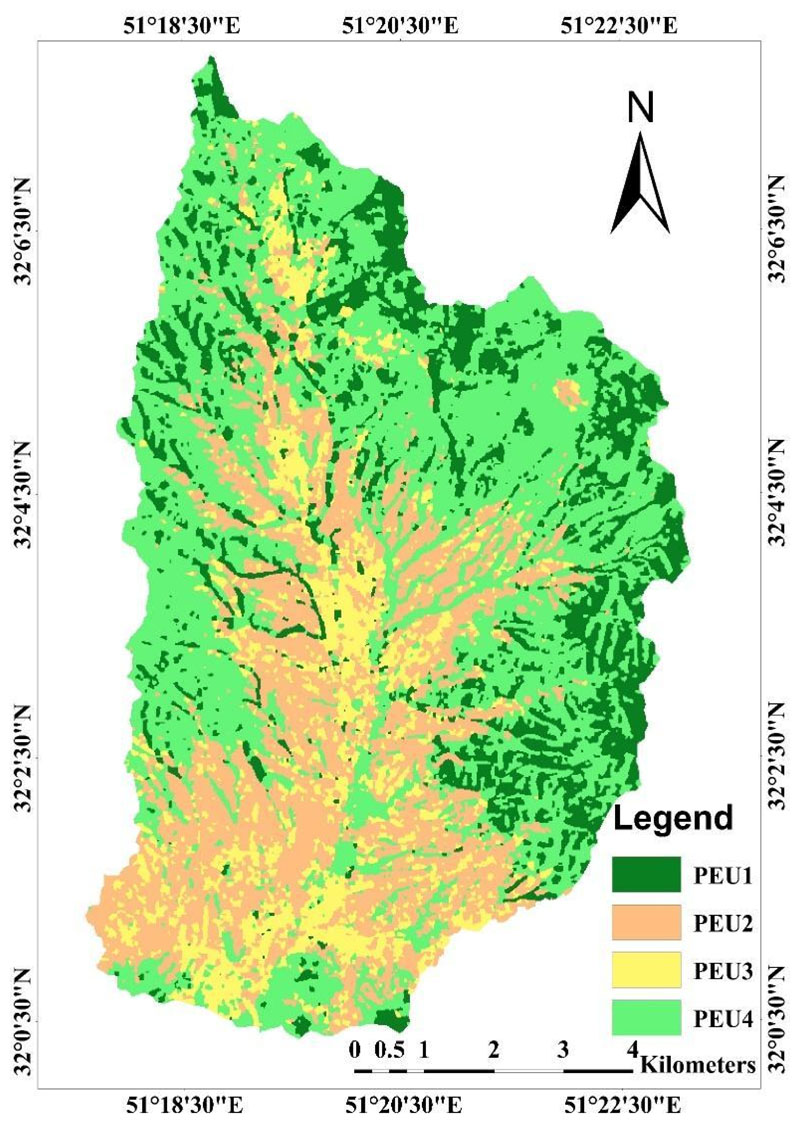
Result of the most accurate PEUs map classifications obtained from Classification Tree Analysis (CTA) algorithm and auxiliary data (PCA) using object-based classification method.

Figure 7 illustrates the most accurate map resulting from raw bands and auxiliary data PCAs using object-based classification that produced the highest overall Kappa accuracy (85%) and overall accuracy (89%). In this map PEU4 is a combination of Shrub - Tallgrass species that accounted for 45% of the entire study area. While PEU3 is a semishrub species that covers little area, accounting for only 12%. PEU1 are shrub species and PEU2 are tallgrass species that accounted for 17% and 24%, respectively.

### 3.5 Statistical Comparison

Based on the “Friedman test with the corresponding post-hoc” the Producer Accuracy, User Accuracy and Kappa index of agreement for PEUs Classes were calculated (Table 4). Likewise, the comparison of the classifier algorithms used in pixel based and objectbased PEUs classification methods are illustrated in Table 1. We used the Friedman test to examine whether the differences between the different classifier algorithms are significant. For the UA and KIA, statistically significant (sig < 0.05) differences appeared between PEUs classes. In addition, the PA is marginally (close to) significant.

## 4 Discussion

The construction of an accurate, fast and simple model for extracting land cover information and vegetation maps is of concern to natural resources managers and ecologists. With the purpose of accurately classifying PEUs across heterogeneous rangelands at the landscape level, four different classifier algorithms were evaluated using pixelbased and object-based approaches. The classification scheme used for separating PEUs is based on the premise that each class is the result of a distinct combination of index species. These index species are the compositional and ecological factors used to separate the PEUs classes. We started with the four dominant PEU classes that account together of the study area: PEU1 (Astragalus verus Olivier (As ve)), PEU2 (Bromus tomentellus Boiss (Br to)), PEU3 (Scariola orientalis Sojak (Sc or)) and PEU4 (Astragalus verus Olivier—Bromus tomentellus Boiss (As ve—Br to). Extraction of PEUs maps is challenging due to the complex spatial structure of the landscape and similar spectral behavior. The OLI sensor images have already demonstrated their utility for vegetation mapping due to its high temporal and spatial resolution and temporal resolution and high quantization [32]. Nevertheless, in our region, the OLI spatial resolution of 30 m likely reflects numerous mixed pixels and consequently low precision specifically at subclass of rangeland land-cover i.e., PEUs. In an attempt to circumvent this mixed-pixels problem, the images were pan-sharpened using panchromatic band (band 8) to improve the spatial resolution of the bands.

### 4.1 The Selection of Pixel-Based and Object-Based Approaches in PEUs Classification

Based on four classification methods applied to PEUs mapping using the Landsat 8 pan-sharpened bands, it was revealed that object-based methods clearly outperformed pixel-based methods. Similarly, previous studies (e.g., [19,33]) have demonstrated that object-based classification methods can provide superior classification accuracy than pixel-based classification methods. As shown in Figure 3 the classification maps produced by the pixel-based classification method presented a noticeable “salt-and-pepper” appearance. In the pixel-based classification method, many of the pixels were assigned to classes that were different from those of the adjacent membership, and usually exploit only spectral information. Therefore, the PEUs showed nonhomogeneous coverage and were characterized by a salt-and-pepper effect that makes it difficult to separate PEUs classes. Conversely, the object-based classification methods were less exposed to these problems. Classified PEUs based on this method had distinct partition zones of PEUs and provided PEU class uniformity (Figure 4). In the object-based approach, the optimization of segmentation parameters, especially the scale parameter, improved the accuracy of the classification map [34].

### 4.2 Selection Best Classification Algorithm

For the MD, NN-MLP and CTA algorithms, the object-based strategy outperformed the pixel-based one, while the MLC algorithm revealed no significant differences between both strategies. Hence, these results suggest that with MLC both pixel/object-based methods can be used to classify PEUs with respect to overall classification accuracy. Similarly, in the study of Xie [16] it was concluded that when spectral bands were used for classification, MLC outdid machine learning algorithms. However, the CTA algorithm, followed by NN-MLP, led to the most accurate results that were reached with the object-based approach, with improvements of overall accuracy up to 19% and 17%, respectively. Treebased algorithms such as Decision Trees, Gradient Boosting and Random Forest are considered among the most powerful machine learning classifiers. The CTA algorithm, sometimes referred to as decision trees (DT) or classification and regression tree analysis (CART), has shown promise for improving classification accuracy, and has received increasing attention. For instance, [17] found that for landform classification the DT algorithm yielded most accurate results. Likewise, Decision tree analysis, such as CTA, holds advantages over traditional supervised algorithms such as maximum likelihood classification. This algorithm being insensitive to noise in input data generally tends to perform fast, robust and efficient [35]. In addition, the CTA algorithm can perform well at an optimal segmentation scale, and is sensitive to segmentation scale variation. Additionally, it can easily handle missing values and can incorporate continuous variables as well as categorical auxiliary data. It makes sense that the CTA algorithm was evaluated as the most reliable method for PEUs discrimination. In addition, here the CTA algorithm showed an obvious preference towards object-based classification strategies for PEUs classification. At the same time, probably further gain in accuracy can be achieved when moving towards Random Forest (RF). RF is an automated iteration of CTA, i.e., it generates multiple decision trees for developing the classification model. This algorithm is especially powerful for situations when having a large dataset available, and the interpretability of the model is not a major concern [2].

### 4.3 Impact of Auxiliary Data on PEUs Classification Accuracy

The second model is based on auxiliary data along with raw bands to obtain the most accurate map of PEUs. PCAs is a linear transformation method, which is valuable in improving PEUs classification accuracy. The results of the second stage (Table 3) showed combinations of PCA with raw bands can improve overall classification accuracy. The comparison of the performance of raw bands and auxiliary data showed that auxiliary data improved overall accuracy 3% and overall kappa of 5%.

The purpose of PCA is to reduce the number of dimensions by gathering the most useful variations. When considering the first three PCAs, more than 99% of spectral information is presented, thereby reducing noise and therefore improving classification accuracies [36]. Likewise, it seems that these auxiliary data compensate for the effects of bare soil reflectance on the received signals by the sensors of the raw bands and presents more pure pixels associated to PEUs. PEU1 benefited most from auxiliary data (10%). In the study area PEU1 is a shrubby species (As ve) and distributed in more steep slopes (Figure 7). Due to their higher canopy cover and consequently delivering pure pixels, therefore, spatial distribution of PEU1 was improved. So, PEU1 was well extracted, especially in the object-based CTA algorithm and auxiliary data. PEU2 is a tallgrass species (Br to) and distributed mainly in the flat areas. As shown in Table 3, PEU2 is the most difficult class to identify due to its wide and complex presence in the vegetation structure. Meanwhile, the highest classification accuracy of the PEU2 with 81% UA was well portrayed by the object-based CTA algorithms and auxiliary data. PEU3 are semi-shrub species that cover little areas (Figure 7). PEU3 is dominated by semi-shrub species (Sc or) that cover little areas. This class is characterized by irregular and sparse distribution with areas of bare soil frequently visible between plants ranging from a few square centimeters to several square meters to even some meters in some cases. Ultimately, sensors record the reflectance of a mixture of vegetation and soil, and the error rate increases in this PEU. This class benefited by including PCA (up to 4% UA). Likewise, the highest classification accuracy of the PEU3 with 85% UA was well portrayed by the object-based CTA algorithms and auxiliary data. It seems that these auxiliary data compensate for the effects of bare soil reflectance on the received signals by the sensors of the raw bands and presents more pure pixels of these PEUs. PEU4 is the combination of shrub-tallgrass species (As ve—Br to) and occurs evenly almost throughout the whole study area. This class, by the objectbased classification with CTA algorithm, had the highest classification accuracy with 95% accuracy. As shown in Figure 7, PCA negatively affects PEU4. This class consists of two dominant species, having dissimilar spectral behavior due to life-form differences; also, PEU4 occurs evenly almost throughout the whole study area, therefore, causing more pixels that are mixed. The PCA components cannot detect and eliminate these errors and were mostly negative for the classification of PEU4. So far, most of the land classification process has been implemented on the main land cover classes using medium resolution images e.g., Macintyre [1] with OA, 50–74%, Pflugmacher [24] with OA, 75.1%, Feng [23] with OA, 76%, Isabel [17] with OA, 72%. In these studies, thanks to the existence of phenomena with distinct spectral behaviors identification and isolation of various phenomena have been achieved with high accuracy and led to accurate classification maps.

Here, the similar spectral behavior of PEUs species in the heterogeneous rangelands complicated the accurate identification of the PEUs boundaries. Subclasses of a land cover, such as PEUs as a subclass of rangeland cover, are more spectrally similar than that of a higher hierarchical land cover, especially when medium spatial resolution imagery like Landsat OLI-8 data is used for mapping. Furthermore, since this study was conducted in a relatively arid region, surface reflectance values of PEUs are often mixed with information of background soil reflectance. Bare soil reflectance imposes a significant impact on the spectral behavior of PEUs characterized by sparse vegetation cover, and usually hides the spectral responses of patches of vegetation covers. Likewise, in a related study about land use/cover classification in arid rangelands, Genbatu Ge [37] observed that rangelands were composed of herbs and shrubs with high spectral similarity, which complicated the classification result. Another remark is that we only exploited reflectance from a single date Landsat image with overall classification accuracies of 89%, suggesting that improvements can be obtained when including temporal data. For instance, Stumpf [38] emphasized that seasonal time-series images contain the temporal aspects of natural phenomena on the grassland surface, and are beneficial for discriminating different land cover types and monitoring vegetation dynamics. Finally, also geographical location plays a role in the accuracy of PEUs classification. The ecological factors of the plant system will be obviously different due to changes in soil, climate and topography. It implies that the here developed classification models may not be directly transferable to other regions.

### 5 Conclusions

Our analysis provides insights into the way advanced classification algorithms can identify heterogeneous vegetation communities with similar spectral behavior and a complex landscape structure. The presented results underline that in a heterogeneous landscape accurate PEUs mapping is determined according to the type of supervised classification, pixel-based or object-based methods, and the choice of the classifier algorithm. Four classification algorithms applied to PEUs classification revealed that the results of the classification algorithms varied considerably, so that the CTA algorithm yielded the most accurate results, followed by MLC and NN-MLP. In addition, the object-based methods clearly outperformed the pixel-based ones. Not all spectral bands offered valuable information in the classifications. Merging auxiliary PCA bands derived from the original Landsat imagery together with the raw bands offered the most valuable information to distinguish PEUs, with an overall accuracy of 89%. The development of a comprehensive classification procedure and deep learning algorithms may stimulate new research directions for accurate PEUs mapping.

## Figures and Tables

**Table 1:** The identified PEUs and their vegetational characteristics in the study area.

Code	Dominant Species^*^	Field Photos	Abbreviation	Structure	Accompanied Species^*^	Dominant Soil Type
PEU1	*Astragalus verus* Olivier. (23.4%)	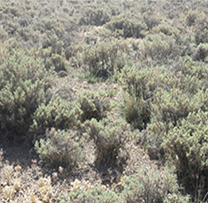	As ve	Scrubby	*Alyssum linifolium Steph*. ex Wild. (2.5%) *Echinophora platyloba DC*. (2.5%) *Scariola orientalis* (Boiss)Sojak. (2.5%) *Eurotia ceratoides* (L.) C.A. Mey. (2%) *Heteranthelium piliferum Hochst*. ex Jaub. (1.8%) *Cousinia bachtiarica* Boiss. & Hausskn. (1.8%) *Bromus tectorum* L. (1.6%) *Astragalus macropelmatus* Bunge. (1.3%) *Taeniatherum crinitum* (Schreb.) Nevski. (1%) *Acanthophyllum spinosum* (Desf.) C.A.Mey. (0.8%)	Sandy loamy to loamy clay
PEU2	*Bromus tomentellus* Boiss. (8.9%)	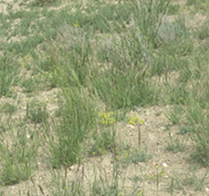	Br to	Grassland	*Phlomis olivieri* Benth. (3%) *Bromus danthoniae* Trin. (3%) *Stipa hohenackeriana* Trin & Rupr. (2.6%) *Alyssum marginatum* Steud. (2.5%) *Bromus tectorum* L. (2.4%) *Achillea wilhelmsii* C. Koch, L. (1.8%) *Astragalus microcephalus* Willd. (1.5%) *Centaurea aucheri* (DC.) Wagenitz. (1.2%) *Gypsophila struthium*. (1%) *Ajuga chamaecistus* Ging. (0.5%)	loamy and Silty Loamy
PEU3	*Scariola orientalis* (Boiss.) Sojak. (9.25%)	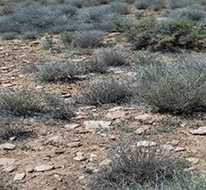	Sc or	Semi-scrub	*Noaea mucronata* (Forsk.) Aschers et. Sch. (2.5%) *Onobrychis cornuta* (L.) Desv. (1.6%) *Astragalus microcephalus* Willd. (1.5%) *Polygonum aridum* Boiss. & Hausskn. (1.5%) *Taeniatherum crinitum* (Schreb.) Nevski. (1.5%) *Cousinia crispa*, Jaub & Spach. (1.2%) *Stachys inflata* Benth. (1.2%) *Tragopogon longirostris* Bischoff ex Sch.Bip. (1%) *Acanthophyllum spinosum* (Desf.) C.A.Mey. (0.5%) *Chardinia orientalis* (L.) Kuntze. (0.5%)	Clay loam
PEU4	*Astragalus verus* Olivier (8.6%)*—Bromus tomentellus* Boiss (5.4)	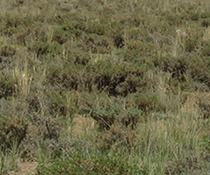	As ve-Br to	Scrubby-grassland	*Noaea mucronata* (Forsk.) Aschers et. Sch. (2%) *Alyssum marginatum* Steud. (1.5%) *Euphorbia azerbadjhanica* Bordz. (1.5%) *Phlomis persica* Boiss. (1.5%) *Turginia latifolia* (L.) Hoffm. (1.5%) *Astragalus effusus* Bunge. (1.3%) *Bromus danthoniae* Trin. (1.2%) *Stachys lavandulifolia* Vahl. (1%) *Cichorium intybus* L. (0.5%) *Achillea wilhelmsii* C. Koch, L. (0.5%)	loamy and Silty Loamy

* Canopy cover percentage of dominant and accompanied species that was calculated on transects.

**Table 2:** Classification accuracies of PEUs using different classification algorithms.

		MD			MLC			NN-MLP			CTA		
	Type	PA	UA	KIA	PA	UA	KIA	PA	UA	KIA	PA	UA	KIA
PBCM
	PEU1	89	80	73	89	91	87	97	67	55	80	86	80
	PEU2	49	67	55	72	79	70	97	49	31	63	64	51
	PEU3	45	36	13	85	67	55	5	100	100	67	58	43
	PEU4	41	47	28	71	84	78	23	5	33	57	61	48
	OK = 42% OA = 57%				OK = 70% OA = 78%			OK = 39% OA = 55%			OK = 55% OA = 67%		
OBCM
	PEU1	89	84	77	89	93	90	93	91	88	97	90	86
	PEU2	63	61	47	72	75	65	69	58	43	93	80	72
	PEU3	32	34	100	83	64	51	65	50	32	83	81	73
	PEU4	50	52	35	69	86	81	41	90	86	69	96	95
	OK = 44% OA = 59%				OK = 71% OA = 78%			OK = 61% OA = 72%			OK = 80% OA = 86%		

Classification algorithms. MD: Minimum Distance, MLC: Maximum Likelihood Classification, (NN-MLP) Neural Network-Multi Layer Perceptron, CTA: Classification Tree Analysis. Methods of classification: PBCM: Pixel-based Classification Method, OBCM: Object-based Classification Method, PA%: Producer’s Accuracy, UA%: User’s Accuracy, KIA%: Kappa Index of Agreement, OA%: Overall Accuracy, and OK%: Overall Kappa.

**Table 3:** Confusion matrix. Summary of classification accuracy for each PEU by raw bands and auxiliary data.

**Accuracy Assessment Results Based on Raw Bands Using Classification Tree Analysis**							
**Type**	**PEU1**	**PEU2**	**PEU3**	**PEU4**	**PA**	**UA**	**KIA**
PEU1	44	0	0	5	97	90	86
PEU2	0	42	8	3	93	80	72
PEU3	0	3	37	6	83	81	73
PEU4	1	0	0	30	69	96	95
	Overall Kappa: 80%				Overall Accuracy: 86%		
**Accuracy Assessment Results Based on Raw Bands + PCA Using Classification Tree Analysis**							
**Type**	**PEU1**	**PEU2**	**PEU3**	**PEU4**	**PA**	**UA**	**KIA**
PEU1	44	0	0	0	97	100	100
PEU2	0	42	6	4	93	81	74
PEU3	0	3	37	4	83	85	78
PEU4	1	0	2	36	82	92	89
	Overall Kappa: 85%				Overall Accuracy: 89%		

Auxiliary data. PCA: Principal Component Analysis, PA: Producer’s Accuracy %, UA: User’s Accuracy %, and, KIA: Kappa Index of Agreement %.

**Table 4:** Results of statistically significant comparison between PEUs accuracy and classifier algorithms accuracy.

PEUs Accuracy	Sig			
		Classification Algorithms	PBCM-Sig	OBCM-Sig
User’s Accuracy (UA)	0.021 *
		MLC-MD	0.036	0.03
		MLC-NN	0.071	0.34
		MLC-CTA	0.22	0.66
		MD-NN	0.77	0.22
		MD-CTA	0.38	0.009
		NN-CTA	0.56	0.17
Kappa Index of Agreement (KIA)	0.039 *
		MLC-MD	0.043	0.030
		MLC-NN	0.083	0.38
		MLC-CTA	0.24	0.66
		MD-NN	0.77	0.19
		MD-CTA	0.38	0.009
		NN-CTA	0.56	0.19
Producer’s Accuracy (PA)	0.095			

The symbol “*” indicates that the difference is statistically significant, because the significant level is 0.05.
